# Novel MRI deformation-heterogeneity radiomic features are associated with molecular subgroups and overall survival in pediatric medulloblastoma: Preliminary findings from a multi-institutional study

**DOI:** 10.3389/fonc.2022.915143

**Published:** 2022-12-21

**Authors:** Sukanya Iyer, Marwa Ismail, Benita Tamrazi, Ralph Salloum, Peter de Blank, Ashley Margol, Ramon Correa, Jonathan Chen, Kaustav Bera, Volodymyr Statsevych, Mai-Lan Ho, Pranjal Vaidya, Ruchika Verma, Debra Hawes, Alexander Judkins, Pingfu Fu, Anant Madabhushi, Pallavi Tiwari

**Affiliations:** ^1^ Department of Biomedical Engineering, Case Western Reserve University, Cleveland, OH, United States; ^2^ Department of Radiology and Biomedical Engineering, University of Wisconsin-Madison, Madison, WI, United States; ^3^ Department of Pathology, Children’s Hospital Los Angeles, Los Angeles, CA, United States; ^4^ Division of Hematology, Oncology & Bone Marrow Transplant, Nationwide Children’s Hospital, Columbus, OH, United States; ^5^ Division of Oncology, Cincinnati Children’s Hospital Medical Center, Cincinnati, OH, United States; ^6^ Department of Neuroradiology, Imaging Institute, Cleveland Clinic, Cleveland, OH, United States; ^7^ Department of Radiology, Nationwide Children’s Hospital, Columbus, OH, United States; ^8^ Department of Population and Quantitative Health Sciences, Case Western Reserve University, Cleveland, OH, United States; ^9^ Radiology and Imaging Sciences, Biomedical Informatics (BMI) and Pathology, Georgia Institute of Technology and Emory University, Atlanta, GA, United States

**Keywords:** medulloblastoma, deformation, molecular subgroups, survival, LASSO

## Abstract

**Introduction:**

Medulloblastoma (MB) is a malignant, heterogenous brain tumor. Advances in molecular profiling have led to identifying four molecular subgroups of MB (WNT, SHH, Group 3, Group 4), each with distinct clinical behaviors. We hypothesize that (1) aggressive MB tumors, growing heterogeneously, induce pronounced local structural deformations in the surrounding parenchyma, and (b) these local deformations as captured on Gadolinium (Gd)-enhanced-T1w MRI are independently associated with molecular subgroups, as well as overall survival in MB patients.

**Methods:**

In this work, a total of 88 MB studies from 2 institutions were analyzed. Following tumor delineation, Gd-T_1w_ scan for every patient was registered to a normal age-specific T_1w_-MRI template via deformable registration. Following patient-atlas registration, local structural deformations in the brain parenchyma were obtained for every patient by computing statistics from deformation magnitudes obtained from every 5mm annular region, 0 < d < 60 mm, where d is the distance from the tumor infiltrating edge.

**Results:**

Multi-class comparison via ANOVA yielded significant differences between deformation magnitudes obtained for Group 3, Group 4, and SHH molecular subgroups, observed up to 60-mm outside the tumor edge. Additionally, Kaplan-Meier survival analysis showed that the local deformation statistics, combined with the current clinical risk-stratification approaches (molecular subgroup information and Chang’s classification), could identify significant differences between high-risk and low-risk survival groups, achieving better performance results than using any of these approaches individually.

**Discussion:**

These preliminary findings suggest there exists significant association of our tumor-induced deformation descriptor with overall survival in MB, and that there could be an added value in using the proposed radiomic descriptor along with the current risk classification approaches, towards more reliable risk assessment in pediatric MB.

## 1 Introduction

Pediatric Medulloblastoma (MB) is an aggressive Grade IV cancer with heterogeneous patient outcomes (1). Current treatment strategies for older children require multimodal therapy inclusive of surgical resection, chemotherapy, and craniospinal irradiation, while deferring radiation strategies for children <3yrs of age (2). These treatment regimens, while effective in achieving long-term survival in a third of MB patients, are associated with short- and long-term morbidities that result from radiation and chemotherapy. For instance, chemotherapy for MB is associated with sensorineural hearing loss and cardiac toxicity (3). Additionally, severe endocrinopathies, bone development deficiencies, and ovarian failure have been reported as side effects of radiation therapy in pediatric MB patients (3, 4). Consequently, many survivors suffer from impaired quality of life, secondary sequelae of radiation injury, and are prone to increased risk for secondary malignancies (5). Better early risk stratification methods may help tailor therapy in MB patients and reduce the impact of therapy for low-risk patients.

Over the last decade, there has been international consensus (6) on the genomic characterization of MB into four distinct molecular subgroups: Sonic Hedgehog (SHH), Wingless (WNT), Group 3, and Group 4 (7). The WNT and SHH sub-groups are named after the signaling pathways that are thought to play prominent roles in the pathogenesis of these two subgroups, while Group 3 and 4 subgroups have been designated generic names since the underlying biology driving them is still not well understood (6). Interestingly, few studies have reported that the four molecular subgroups generally demonstrate different anatomical origins at diagnosis. For instance, Gibson et al. (8) have shown that WNT subgroup is located within the IV ventricle towards the brainstem, unlike SHH subgroup that tends to be far from the brainstem within the cerebellar hemispheres, which could be indicative of the growth patterns of these subgroups as well as the neurological deficits associated with each (9). Additionally, the four MB molecular subgroups have generally shown different clinical behaviors and may benefit from subgroup-specific treatments and targeted therapies (6). Considering these observations, there are ongoing clinical trials to de-escalate therapies for the subgroups considered less aggressive (WNT, SHH), while escalating therapies for the ones that are considered more aggressive (Group 3, Group 4) (6). Interestingly, Cavalli et al. (10) have recently highlighted the presence of intertumoral heterogeneity within the four medulloblastoma subgroups by delineating the presence of 12 additional subtypes across the four MB subgroups. However, despite these developments, the degree of heterogeneity and the extent of overlap within molecular subgroups, as well as their role in treatment modification, are still areas of active investigation in MB tumors.

Diagnosis, surgical guidance, and post-treatment follow-up response assessment in MB tumors is currently investigated using multi-parametric Magnetic Resonance imaging (MRI) including gadolinium-based contrast agent (Gd-T_1w_) sequences. Recent studies have shown the promise of radiomic (high throughput feature extraction) features extracted from routine MRI scans in capturing the underlying tumor histological and molecular characteristics in MB (11, 12) as well as adult brain tumors (13, 14). Hence, there may be an opportunity to complement existing molecular strategies with radiomic markers extracted from routine MRI scans, to further improve clinical patient stratification prior to surgery, allowing for neo-adjuvant subgroup specific therapy, or inform the need for aggressive surgical resection.

MB tumors are known to grow heterogeneously and may impact the neighboring structures, when the growing tumor displaces the surrounding healthy tissue structures (a phenomenon known as mass effect). Hence, the impact of the tumor is observed not just within the visible tumor boundaries, but also in the immediate peri-tumoral, as well as in seemingly normal-appearing adjacent regions (i.e., brain around tumor (BAT). For instance, Group 3 tumors that have relatively poor survival outcomes, are shown to proliferate at a faster rate than WNT subgroup which is associated with improved prognosis (15–17), suggesting that aggressive tumor phenotypes may exert increased tissue distortion on BAT region, compared to the less aggressive ones (e.g., WNT). On Gd-T_1w_ MRI, this exerted tissue distortion may be quantified as local structural deformations in the brain parenchymal region within the vicinity of the tumor.

**Figure 1:**
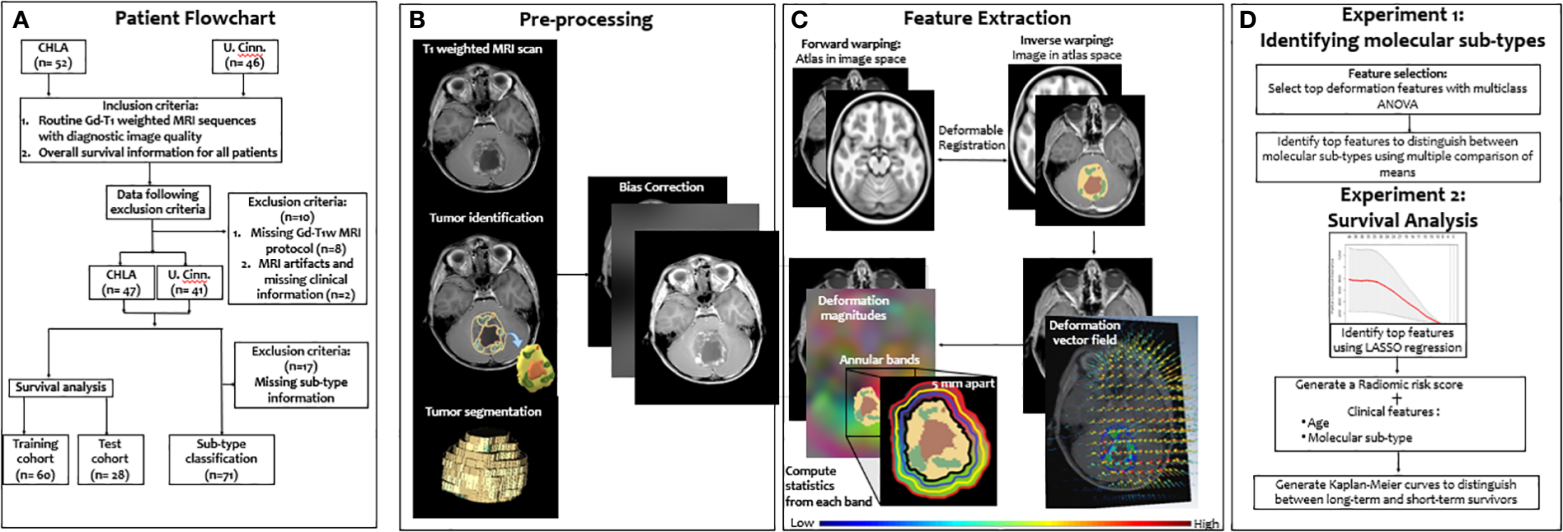
Overview of the proposed framework. A breakdown of our datasets is provided **(A)**. Then, segmentation of tumor compartments and the preprocessing pipeline are shown **(B)**. Deformation features that characterize the tumor impact on BAT region are then extracted **(C)**. Features were then used to 1) identify the 4 molecular subgroups using ANOVA, and 2) survival risk-stratification using a LASSO model for stratifying MB patients into low- and high-risk groups based on their overall survival **(D)**".

In this work, we present a novel deformation-based radiomic descriptor that captures local structural deformations in the BAT region of pediatric MB patients on routine Gd-T_1w_ MRI scans. Our work is based on the rationale that the differential growth patterns across MB molecular subgroups (due to their varying degrees of malignancy) could be quantified using the local structural deformations in the BAT region and may exhibit distinct deformation patterns across the subgroups. Specifically, we hypothesize that (1) the growth pattern of the more aggressive and heterogeneous MB tumors will exert more pronounced structural deformations on the surrounding BAT regions, as compared to less aggressive MB tumors, and (2) these distinct local deformation attributes from the normal brain parenchyma, as captured on Gd-T_1w_ MRI, will be independently associated with the four molecular subgroups (that vary in the degree of aggressiveness to some extent), as well as overall survival in MB patients. **Figure 1** shows the pipeline of the entire framework.

## 2 Methods

### 2.1 Study population

Our retrospective study utilized Gd-T1w MRI sequences from pediatric MB patients from two collaborating institutions. The cohort from Children’s Hospital of Los Angeles (CHLA) consisted of 52 studies (mean age = 5.46 years) and the cohort from Cincinnati Children’s Hospital Medical Center (CCHMC) consisted of 46 studies (mean age = 5.28 years). The sequences used in this study were acquired using both 1.5 and 3 Tesla scanners using the gradient recalled (GR) echo sequence. **Table 1** includes additional details on data acquisition and scanner information. We further triaged studies based on the inclusion criteria (**Figure 1**) to only include Gd-T_1w_ MRI sequences with (1) acceptable diagnostic quality (as identified by collaborating radiologists), and (2) available overall survival information. Specifically, a total of 10 cases were excluded from the analysis; one case was excluded due to poor image quality during acquisition, one was excluded due to the lack of clinical information, and the other 8 cases were excluded because of the missing protocols that were needed to conduct the analysis (axial T1-weighted post contrast scan). This ultimately resulted in a cohort of n=88 cases (47 from CHLA and 41 from CCHMC), of which n=71 cases had the subgroup information available (SHH, WNT, Group 3, Group 4). **Table 1** provides details on the dataset demographics as well as a breakdown of the training and test cohorts employed in this study. Additionally, in supplementary documentation, we have provided the clinical information for the participating cohorts, including Chang risk stratification at diagnosis, molecular subgroup, extent of surgical resection (EOR), and presence of metastatic disease. Further, the original treatment protocol, craniospinal radiation doses, and information on proton radiation at diagnosis are also provided for each patient.

**Table 1:** (A) Data distribution across training and test cohorts. (B) Patient demographics (age, subgroup, and survival information) as well as scanner information.

A	Data Distribution	
Site	Training cohort(n=60)	Test cohort(n=28)
CHLA	32	15
CCHMC	28	13
Total cases	60	28
B	Demographics and Scanner information	
Parameter	Training cohort (n=60)	Test cohort (n=28)
Age (mean, years)	5.462	5.2895
OS (mean, days)	1573	1384.7
Subgroup:		
WNT	4	3
SHH	15	6
Group 3	8	3
Group 4	22	10
Group 3/Group 4	6	3
Missing	5	3
Scanner: 1.5 T Philips Acheiva scanner at CHLA	32	15
Scanner: 3 T Philips Ingenia scanner at CCHMC	28	13
Scan type	T1 FFE axial post-contrast *	T1 FFE axial post-contrast *
MR acquisition type	2D	2D
Scanning sequence	Gradient recalled (GR)	Gradient recalled (GR)
Sequence variant	Steady state (SS)	Steady state (SS)
Pixel spacing (mm)	0.46-1	0.46-1
Slice thickness (mm)	1-5.8	1-5.8

*T1 Fast Field Echo axial post-contrast.

Due to the small and disproportionate number of cases across each of the molecular subgroups from the two institutions (CHLA, CCHMC), we incorporated the entire cohort of n=71 patients (for whom the molecular information was available) for unsupervised statistical analysis, i.e., without holding out a validation set. Further, in our survival analysis experiment, the disproportionate distribution of MB subtypes was accounted for by dividing the entire cohort of n=88 studies into training and test set using a 70-30 split; following the essential caveat that all MB subgroups are proportionally represented across the training and test sets.

### 2.2 Pre-processing

For every study used in this work, Gd-T1w MR images were corrected for inhomogeneities caused by the magnetic field using N4 bias correction, followed by applying a histogram matching method for intensity standardization as described in (18). Specifically, we generated a normalized ‘intensity histogram template’ by randomly selecting 8 patients from our cohort (~10% of the cohort), to generate a template distribution. Then, we computed the intensity histograms for these randomly selected patients and normalized these histograms to obtain a single histogram which represented the template. Distributions for all our patient volumes from the two sites were then nonlinearly mapped to the template distribution using the intensity normalization method as presented in (18), thus bringing all patients into the same intensity range. The T2-w/FLAIR MR images were then employed for annotation of peritumoral edema, following their registration to Gd-T1w MRIs, to ensure alignment. Specifically, two experts performed the manual annotations on every MRI slice, *via* a hand-annotation tool in 3D Slicer. Expert 1 (V.S), a board-certified neuro-radiologist with 10 years of experience, supervised expert 2 (K.B, with 4 years of radiology experience), in annotating the MRI slices into the following 2 regions: (1) infiltrating T2/FLAIR hyperintensities (edema), and (2) the normal adjacent region that exists around the infiltrating tumor margins as BAT.

### 2.3 Feature extraction

To capture structural deformations in the BAT region, all Gd-T1w images of the diseased subject brains were registered to age-appropriate atlases that were obtained from the neurodevelopmental MRI database (19). Specifically, to account for anatomical differences across different age groups due to brain development in pediatric patients, a total of 4 age-specific atlases (0-1, 1-5, 5-10, 10-18 years) were used from this database. A two-step registration scheme was then employed, which is based on diffeomorphic transformation that guarantees symmetry regardless of the chosen similarity measure and generates an inverse mapping (20). In this scheme, the atlases were first non-rigidly registered to the subjects using mutual information-based similarity measure within a B-Spline registration scheme (20). The tumor mask (the infiltrating T2/FLAIR hyperintensities) was removed from the subject brain during registration such that only the spatial intensity differences due to structural deformation caused by mass effect were recovered, when compared to the atlases. This step yields the forward transformation of the voxel-wise deformation field (including the affine components), which maps the displacements of the voxels between the reference and floating volumes. The second step of this registration scheme utilizes the inverse mapping of the diffeomorphic registration that yields the tissue deformations with respect to the atlases, by warping the subjects into the atlas space, to compute subtle per-voxel structural deformations. Per-voxel deformation magnitudes were then computed, by calculating the Euclidean norm of the scalar values of the deformation orientations which resulted from the new voxel positions after mapping each volume to the atlas space.

Finally, to capture localized deformation changes around the infiltrating edge, the region outside the infiltrating T2/FLAIR hyperintensities was divided into equidistant 12 annular bands. These bands were created to be 5mm apart from each other, covering 60 mm outside the tumor margins. The choice for the size of the annular bands was based on previous studies which recommended 5mm as the safe clinical target volume margin for MB tumors (21). The deformation magnitudes of voxels within each of the bands were aggregated and individually analyzed, where each band was inclusive of the previous margin. For each of the 12 bands outside the tumor region, five statistics were obtained from the extracted deformation field for every band; mean (M), median (MD), standard deviation (STD), skewness (SK), and kurtosis (K). These aggregated statistical measures of deformation magnitudes (n = 60 features (i.e., 5 feature statistics across the 12-bands)) were then further pruned to identify independent subsets of local structural deformation attributes that are significantly associated with 1) the four molecular subgroups, and 2) overall survival, in MB patients.

### 2.4 Statistical analysis

*Experiment 1:* Identifying associations of structural deformation features with MB molecular subgroups using ANOVA

In this experiment, we sought to identify if there exist significant associations between the extracted deformation features with the four molecular subgroups of MB (SHH, WNT, Group 3, Group 4). Highly correlated deformation features from across each of the 12 bands were discarded, and the remaining features were scaled and normalized. A multiclass ANOVA test was used to obtain statistically significant differences in deformation features across the subgroups. This was followed by a *post hoc* test, ‘multiple comparison of means’, to identify if the means of each of the subgroups have significant statistical differences from other MB subgroups (22).

Additionally, we conducted a comparative experiment in light of the work by Dasgupta et al. (23), where we extracted semantic features and correlated their impact on molecular subgroups of our MB cohort. Specifically, five semantic features were extracted from all cases that had subgroup information available, including Group 3/Group 4 patients (n=81) across both datasets: (1) tumor’s horizontal position Midline/Lateral, (2) tumor’s vertical position: Superior/Central/Inferior, (3) necrosis: Present/Absent, (4) brainstem involvement: Present/Absent, and (5) contrast uptake: Low/Medium/High. The distribution of semantic MRI features amongst the five molecular subgroups was compared using the Pearson Chi-square test and Fisher’s exact test as appropriate. The Fisher’s exact test was used when a semantic feature had two classes (binomial classification) and the Pearson’s chi-square test was used when a semantic feature had more than two classes (non-binomial classification). The five subgroups (including the transitional G3/G4) were analyzed in a one vs all setting with a 95% confidence interval.

*Experiment 2:* Identifying the added-value of structural deformation features for MB survival risk-stratification in conjunction with existing approaches, and comparison with clinical variables

First, to evaluate the associations of the local deformation features in the context of survival analysis in MB, we employed a least absolute shrinkage and selection operator (LASSO) regression model (24), along with a cox proportional hazards model. We implemented LASSO using the glmnet package in R (25), where only a subset of the features was selected, while forcing the coefficients of other features to be zeroes. The LASSO model was run on the training set using a 3-fold cross-validation scheme to avoid selection bias. A radiomic risk score (RRS) was then generated using the top features selected by the LASSO model, where the weighted sum of these features was used to create a continuous risk score for each patient in both training and test cohorts. Finally, we used the X-tile software (version 3.6.1) to find an optimal threshold using a grid-search across all the risk score values to classify patients into high-risk and low-risk groups in the training cohort. Kaplan–Meier survival analysis was employed to examine the differences in overall survival between the identified low- and high-risk groups.

Additionally, to assess the performance of RRS and the added value of combining it with the current stratification approaches for pediatric MB, we performed the following analyses, for MB survival prognostication: (1) RRS alone, (2) RRS + Chang’s stratification, (3) RRS + molecular subgroup information, and (4) RRS + Chang’s stratification + molecular subgroup information. For our multivariate cox proportional hazards models, we reported multiple hazard ratios as we obtained distinct hazard ratios for each stratum of every independent covariate. Within each covariate, one stratum will have the ‘baseline’ hazard ratio of 1 against which the other strata are compared. A hazard ratio > 1 indicates increased hazard while a hazard ratio <1 indicates a lower hazard than the baseline strata.

Further, for sake of completeness, we utilized the available clinical information for our participating cohorts, to assess their performance in survival prediction on our cohort. Namely, the following parameters were individually employed, i.e., in a univariate setting, into our survival model: (1) Chang’s stratification, (2) molecular subgroup information, (3) tumor volume, (4) EOR, and (5) presence of metastasis.

*Experiment 3: Assessing associations between RRS and existing risk-stratification approaches (Chang, and molecular risk-assessment)*

To assess if there exist any statistically significant relationships between the survival predictions of RRS versus the current stratification approaches, we performed the following experiments: (a) McNemar’s test for correlated proportions on RRS outcomes and Chang’s stratification outcomes (null hypothesis being that the two models are equal), and (b) Chi-square test for independence between RRS and molecular subgroup stratification. For McNemar’s test, a 2 x 2 contingency table was constructed based on the predictions of low-, and high-risk groups *via* both stratification approaches. Similarly, for Chi-square test, a 4 x 2 contingency table was constructed for comparing predictions from each of the 4 molecular sub-types as well as RRS. For both tests, the chi-squared statistic and the associated p-values were reported.

## 3 Results

### 3.1 Experiment 1: Identifying significant associations of structural deformation features with MB molecular subgroups using ANOVA

**Figure 2:**
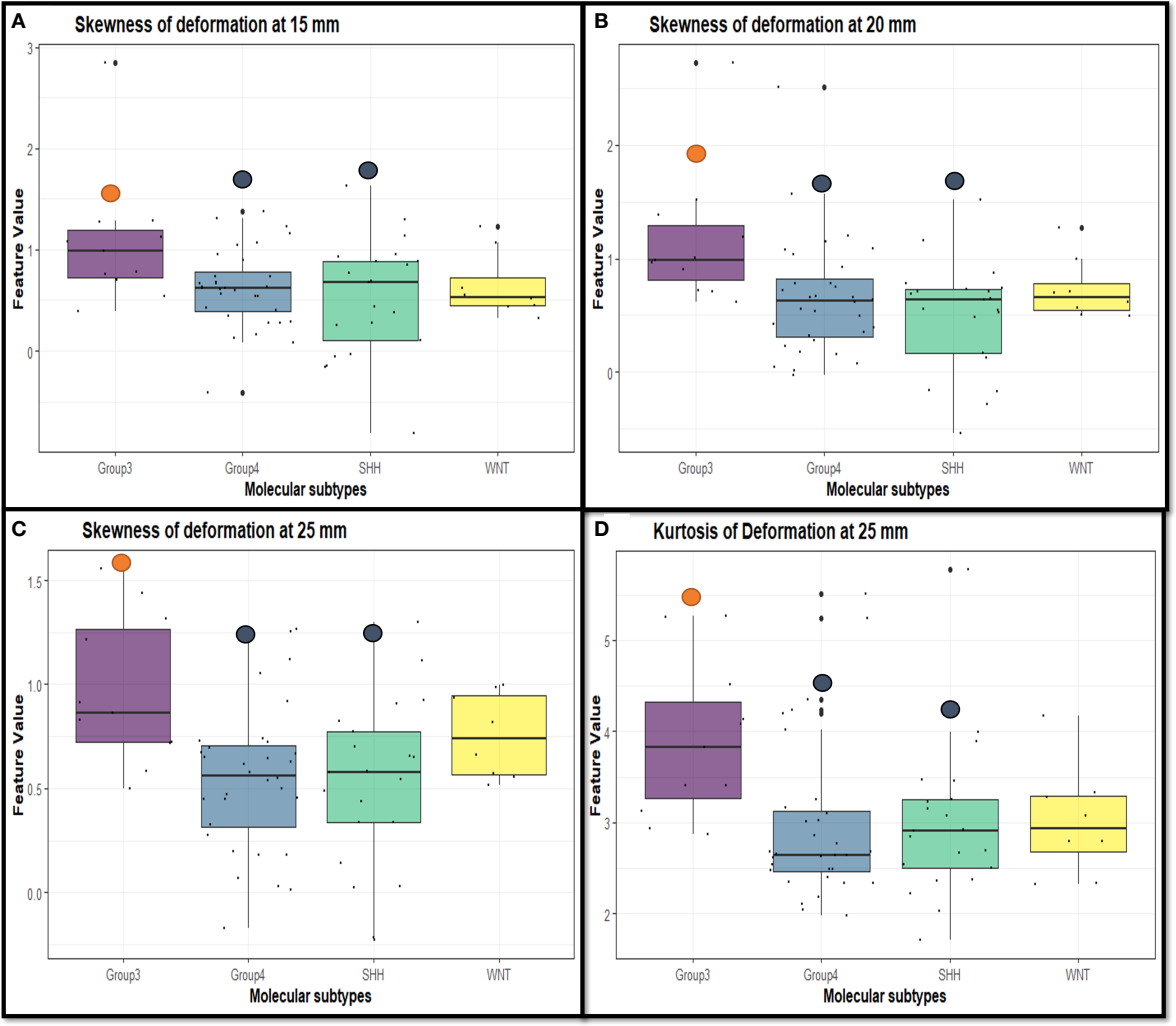
Box plots representing the top deformation features that were found to be discriminative between the MB subgroups. Specifically, skewness of deformation was statistically significant at **(A)** 15 mm between Group 3 vs. SHH and Group 4 (p-value = 0.028), **(B)** 20 mm between Group 3 vs. SHH and Group 4 (p-value = 0.007), and **(C)** 25 mm between Group 3 vs. SHH and Group 4 (p-value = 0.004). Kurtosis of deformation was statistically significant at **(D)** 25 mm between Group 3 vs. SHH and Group 4 (p-value = 0.018).

**Figure 3:**
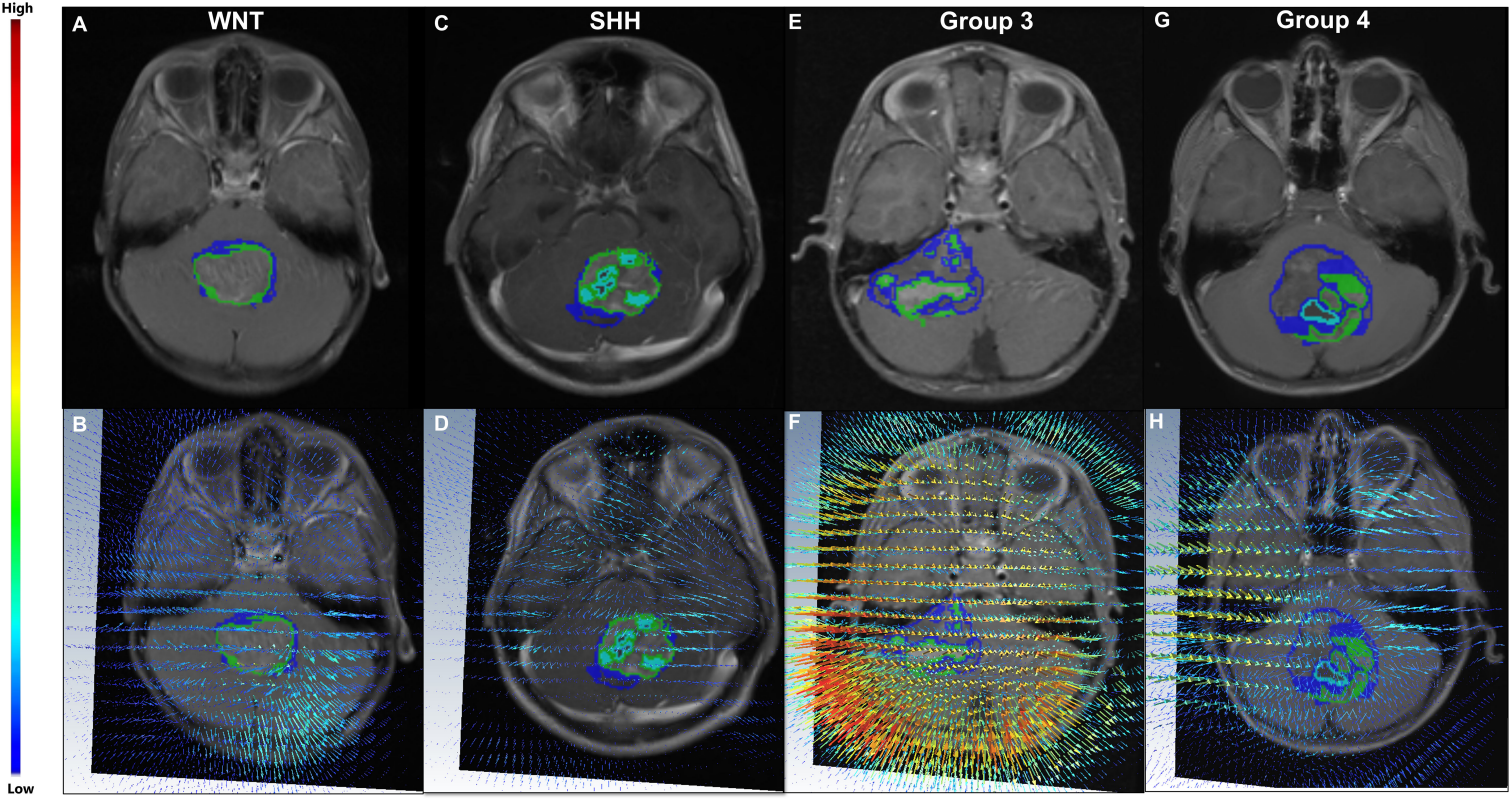
(Top) Four MB subjects representing the four MB molecular subgroups, WNT **(A)**, SHH **(C)**, Group 3 **(E)**, and Group 4 **(G)**, respectively. The corresponding deformation magnitude maps for each of the four molecular sub-types are provided in **(B, D, F, H)** respectively. For the patient with Group 3 (the most aggressive subgroup) **(E)**, higher magnitude values (represented in red) were observed in close proximity of the tumor **(F)**, whereas lower values were observed (blue) in **(B)** for the patient with WNT (the least aggressive subgroup) **(A)**.

**Figure 4:**
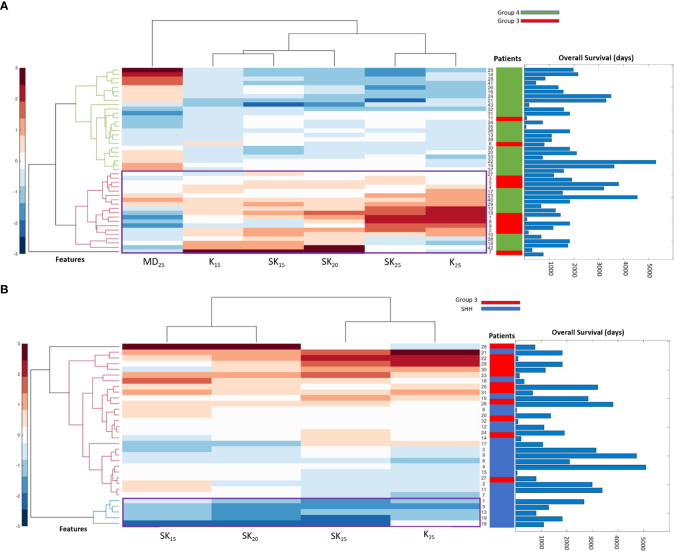
Hierarchical clustering of different MB subgroups based on the conducted ANOVA. In **(A)**, the statistically significant features between Group 3 and Group 4 were used to create a dendrogram that clusters the patients into their subgroups. Overall survival (OS) information for patients is also displayed, where patients clustered into Group 3 have lower OS than those clustered into Group 4. The statistically significant features between SHH and Group 3 are used in **(B)** to create a dendrogram that clusters the patients into their subgroups. OS information is also displayed for each patient, where Group 3 shows worse OS than SHH that is usually associated with better prognosis.

**Table 2** shows the statistically significant deformation features across the 4 molecular sub-types along with their p-values. Interestingly, skewness of deformation magnitudes was found to be statistically significantly different between the molecular subgroups at several distances from the tumor margin, including at 15 mm (SK_15_), at 20 mm (SK_20_), and at 25 mm (SK_25_), between Group 3 vs. SHH and Group 4. Additionally, kurtosis of deformation showed significant differences between the molecular subgroups, where K_15_ was statistically significantly different between Group 3 and Group 4, and K_25_ was statistically significantly different between Group 3 vs. SHH and Group 4. Another top feature was median of deformation, where MD_25_ was statistically significantly different between Group 3 and Group 4. **Figure 2** includes the box plots of the top deformation features among the four MB subgroups. Additionally, **Figure 3** shows the qualitative differences among the four MB subgroups. WNT, the least aggressive subgroup, seemed to exhibit lower deformation magnitude values, whereas more aggressive subgroups (e.g., Group 3 and Group 4) exhibited higher deformation magnitude values. **Figure 4** shows the hierarchical clustering of the different MB subgroups, based on the statistically significant deformation features that resulted from ANOVA.

**Table 2:** ANOVA results for identifying the four molecular MB subgroups.

Deformation features	Molecular subgroups	p-value
Skewness at 15 mm	Group 3 vs. SHH and Group 4	0.028
Skewness at 20 mm	Group 3 vs. SHH and Group 4	0.007
Skewness at 25 mm	Group 3 vs. SHH and Group 4	0.004
Kurtosis at 15 mm	Group 3 and Group 4	0.05
Kurtosis at 25 mm	Group 3 vs. SHH and Group 4	0.0183
Median at 25 mm	Group 3 and Group 4	0.05

Six features showed statistically significant differences among the subgroups.

Our analysis pertaining to the association of tumor location-specific semantic features with molecular sub-types yielded statistically significant association between Group 4 tumors and midline horizontal location (p-value = 0.0034) as well as between Group 4 tumors and presence of brainstem involvement (p-value = 0.0034). Additionally, we identified statistically significant associations between Group 3 tumors and high contrast uptake (p-value = 0.00001), and a statistically significant association between SHH tumors and medium contrast uptake (p-value = 0.000549).

### 3.2 Experiment 2: Identifying the added-value of structural deformation features for MB survival risk-stratification in conjunction with existing approaches, and comparison with clinical variables

**Figure 5:**
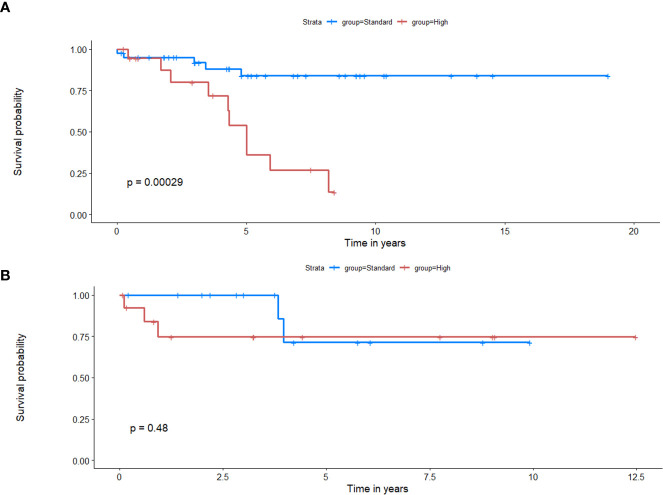
Kaplan Meier curves that illustrate survival analysis conducted using the top deformation features that created the radiomic risk scores (RRS) for both training **(A)** and test **(B)** sets. X-axis represents overall survival in years, and Y-axis represents the estimated survival function.

**Figure 6:**
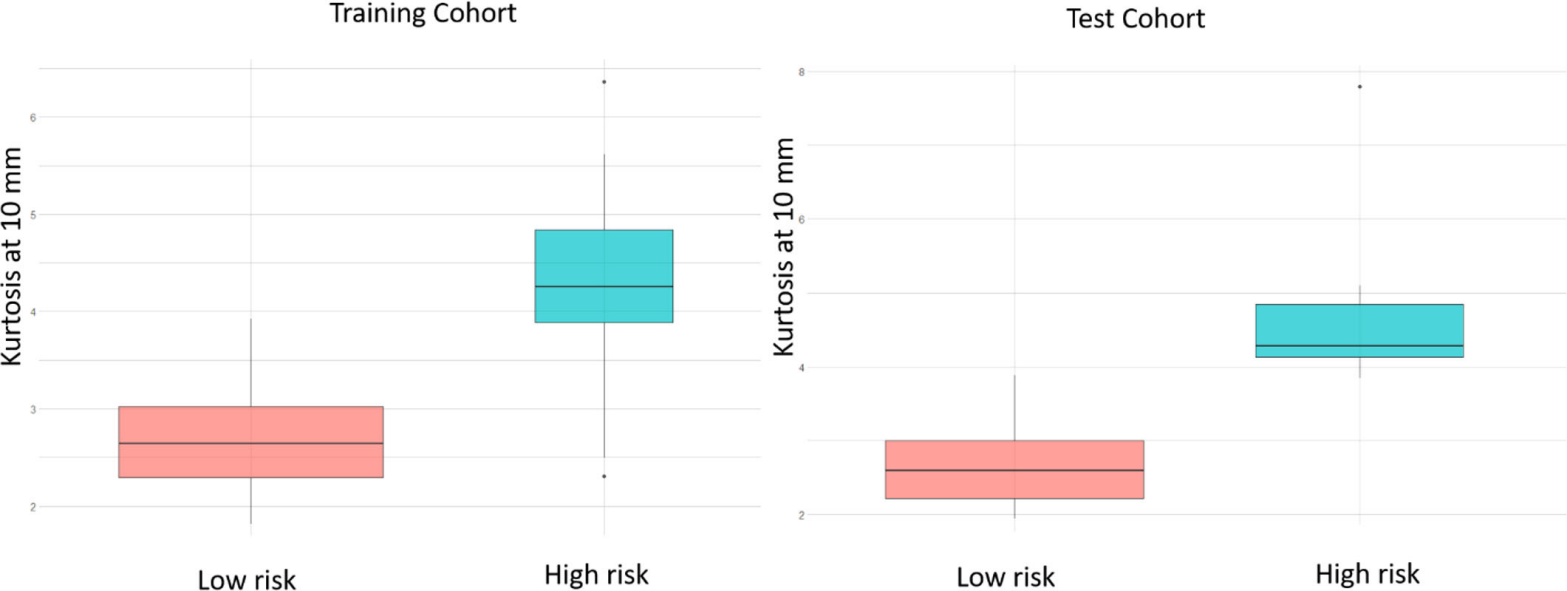
Box plots of the most significantly different deformation feature (kurtosis at 10 mm) between MB patients with poor OS and those with prolonged OS, of the training and test sets respectively, based on the LASSO regression model. p-values for the differences across the 2 risk groups are 0.02 and 0.017 for training and test sets respectively.

*Employing deformation features alone (RRS)*: When applied on the training set, LASSO survival analysis yielded 3 top deformation features (K_10_, K_20_, and MD_25_). The KM curve obtained for the training set based on these features (**Figure 5A**) demonstrated significant differences between the two groups, p-value = 2.9 × 10^-4^. The C-index obtained was 0.7, with a hazard ratio (HR) of 5.9. However, for the test set, significant differences were not observed between the low-risk and high-risk survival groups (**Figure 5B**), perhaps on account of the small testing cohort. Boxplots for the most discriminative deformation feature (K_10_) are shown in **Figure 6** for both training and test sets, where p-values for the training and test sets were found to be 0.02 and 0.017 respectively.

**Figure 7:**
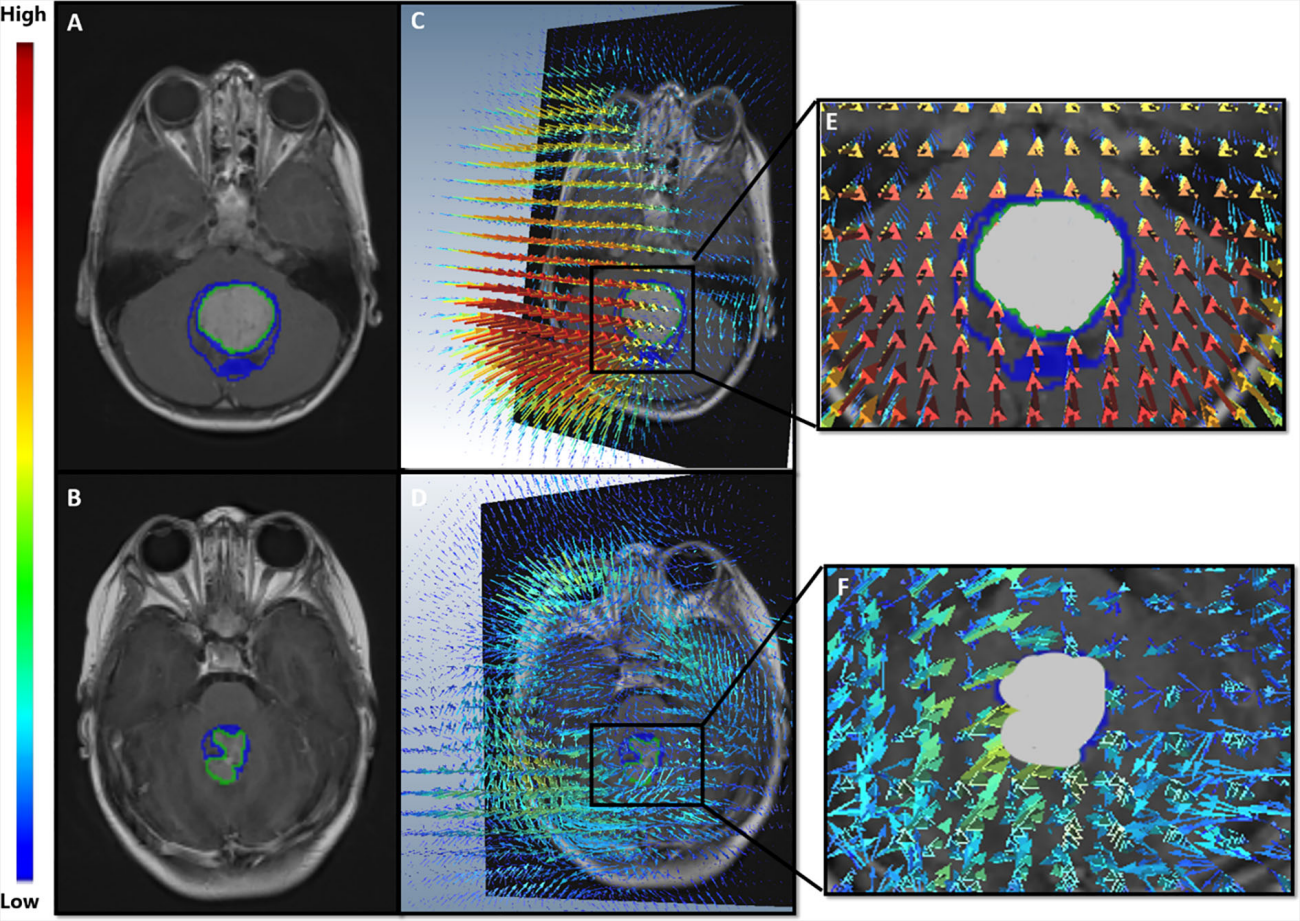
**(A, B)** show Gd-T1w MR scans of the two MB patients; a patient with poor survival (top row), and a patient with prolonged survival (bottom row). **(C, D)** illustrate the extracted deformation field magnitudes for each of the two patients, respectively. For the patient with poor survival **(C)**, higher magnitude values (represented in red) were observed in close proximity of the tumor, whereas lower values were observed (blue) for the patient with prolonged survival **(D)**.

Qualitative differences for the deformation fields for two MB subjects, one with poor-survival (top row) and one with prolonged survival (bottom row), are shown in **Figure 7**. The patient with poor survival seemed to exhibit higher values of the deformation field magnitudes for kurtosis of deformation (kurtosis is a measure of extreme values in a dataset) and seemed to reflect higher values for the patient with worse survival, compared to that for the patient with improved survival.

**Figure 8:**
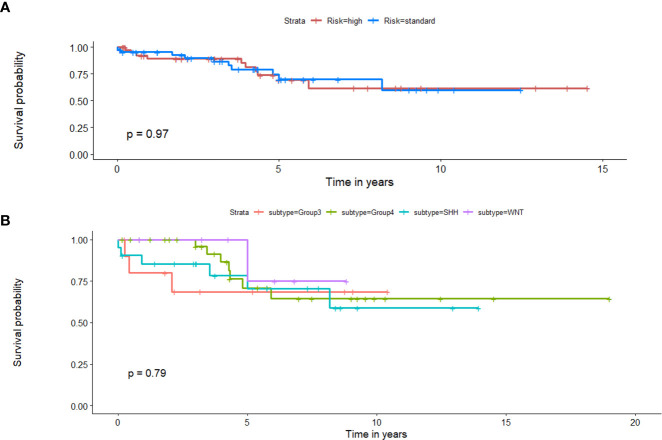
Kaplan Meier curves that illustrate survival analysis conducted using **(A)** Chang’s stratification information, and **(B)** molecular subgroup information for the MB subjects from both datasets. X-axis represents overall survival in years, and Y-axis represents the estimated survival function.

*Employing clinical features in a univariate setting for survival analysis*: When Chang’s classification (n = 88), molecular subgroup information (n = 71), EOR (n =88), presence of metastatic disease (n = 88), and tumor volume (n = 88) were individually employed to survival analysis in a univariate setting, significant differences were not observed between the two survival risk groups. **Table 3** shows the performance metrics for each of these experiments. Additionally, **Figure 8** shows the KM estimates for survival, when employing Chang’s stratification as well as molecular subgroup information, in a univariate setting to prognosticate survival. Additional performance metrics of each of these models are available in supplementary documentation.

**Table 3:** p-value, concordance indices, and hazard ratios for the survival experiments conducted in a univariate setting using Chang’s classification, molecular subgroup information, and the tumor volume information.

Analysis	Feature	Stratum	Entire Cohort		
			HR	C-index	p-value
**Uni-variable**	Chang’s(n = 88)	Standard-risk	1	0.546 ± 0.072	0.97
		High-risk	0.98		
	Molecular subgroup(n = 71)	Group 3	1	0.624 ± 0.064	0.79
		Group 4	0.59		
		WNT	0.48		
		SHH	0.80		
	Tumor Volume(n = 88)	–	1.17	0.55 ± 0.065	0.2

Distinct hazard ratios are reported for each stratum, in the event there is more than one independent covariate in the survival analysis model.

*Employing RRS with clinical features in a multivariate setting for survival analysis*:

(a) RRS + Chang’s stratification: Significant differences were identified between the two risk groups of the training cohort (p-value = 0.002, C-index = 0.695). However, RRS + Chang’s stratification did not yield significant differences between the low-risk and high-risk groups on the test cohort (p-value = 0.7, C-index =0.53).
(b) RRS + molecular subgroup information: Significant differences were observed between the two risk groups of the training cohort using this RRS + molecular subgroup information (p-value = 0.002, C-index = 0.751), demonstrating an improvement in C-index value from the model in (a). While the RRS + molecular subgroup analysis, similarly, did not show significant differences on the test cohort (p-value = 0.4), substantial improvement in the C-index (0.81) was observed on the test set, compared to the values obtained when employing either RRS or molecular information in a univariate setting.
(c) RRS + Chang’s stratification + molecular subgroup information: Significant differences were observed between the two risk groups on the training cohort (p-value = 0.005, C-index = 0.75). While the RRS + Chang’s stratification + molecular subgroup analysis did not significant differences on the test cohort, substantial improvement in the C-index (0.792) was observed on the test set, compared to the values obtained when employing either RRS, molecular information, or Chang stratification, in a univariate setting.

**Table 4** lists Hazard ratios, concordance-index, and the corresponding p-values for all the conducted survival experiments in a multivariate setting.

**Table 4:** p-values, concordance indices, and hazard ratios for the survival experiments conducted using our radiomic risk score (RRS), in both univariate and multivariate settings.

Analysis	Feature	Stratum	Training			Testing		
			HR	C-index	p-value	HR	C-index	p-value
**Univariable**	RRS (n = 88)Training: n = 60Testing: n = 28	Standard-risk	1	0.7 07 ± 0.08	2.9 × 10^-4^	1	0.44 ± 0.1	0.48
		High-risk	5.9			1.9		
	RRS + Chang’s(n = 88)Training: n = 60Testing: n = 28	RRS	2.5	0.695 ± 0.08	0.002	0.82	0.53 ± 0.15	0.7
		Chang’s high-risk	1			1		
		Chang’s standard-risk	1.2			0.47		
**Multivariable**	RRS + molecular information(n = 71)Training: n = 49Testing: n = 22	RRS	2.5	0.761 ± 0.08	0.002	0.82	0.812 ± 0.087	0.4
		Group 3	1			1		
		Group 4	0.2			0.92		
		WNT	0.2			1.3		
		SHH	0.15			2.7		
	RRS + Chang’s + molecular information(n = 71)Training: n = 49Testing: n = 22	RRS	2.57	0.746 ± 0.079	0.005	0.84	0. ± 0.09	0.5
		Chang’s high-risk	1			1		
		Chang’s standard-risk	0.98			0.63		
		Group 3	1			1		
		Group 4	0.23			1.4		
		WNT	0.16			2.1		
		SHH	0.16			3.5		

The univariate model includes RRS only. The experiments conducted in a multivariate setting used a combination of RRS and clinical information such as Chang’s classification and molecular subgroup information. Distinct hazard ratios are reported for each stratum, in the event there is more than one independent covariate in the survival analysis model.

### 3.3 Experiment 3: Assessing associations between RRS and existing risk-stratification approaches (Chang, and molecular risk-assessment)

McNemar’s test for correlated proportions on RRS outcomes and Chang’s stratification outcomes did not yield statistical significance (p = 0.44), suggesting that the predictions of the two models do not hold significant associations between their assessments. Similarly, the chi-squared test between survival predictions of the RRS and molecular sub-group analysis did not yield significant associations (p = 0.86), suggesting that RRS may provide complementary risk-assessment, independent of the existing approaches. The associated contingency tables for the two tests are provided in supplementary documentation.

## 4 Discussion and conclusions

Medulloblastoma (MB) is an aggressive brain tumor in children, that can have both local mass effect as well as potential for metastasis through the CNS. Locally, this results in variable degree of normal brain parenchymal displacement, that can ultimately lead to worse prognosis and poor survival (26). In this work, we presented a deformation-based image descriptor that leverages the structural deformation attributes from the brain around tumor (BAT) regions, with the underlying hypothesis that the growth patterns of more aggressive and heterogeneous MB subgroups will lead to more pronounced structural deformations in the surrounding BAT regions, as compared to less aggressive subgroups. While previous approaches have explored local structural deformations as a radiomic descriptor to prognosticate survival in adult Glioblastoma tumors (13, 14), a common symptom among brain tumors is the elevated intra-cranial pressure (27). Additionally, the study by (28) demonstrated that elevated intra-cranial pressure is a major cause of structural malformations in MB patients. We thus rationalized that it might be reasonable to explore the associations of structural deformations with patient prognosis in the context of MB tumors as well, as these tumors similarly tend to be aggressive and highly malignant. To our knowledge, our work presents the first attempt at exploiting distinct local tissue-induced deformation signatures for identifying associations of deformation features with molecular subgroups, as well as with overall survival, in the context of pediatric MB tumors.

Further, previous studies have employed radiomic and deep-learning approaches to distinguish molecular subgroups of MB using features obtained from within the visible peri-tumor confines alone (11, 29, 30). For instance (11), combined a set of imaging attributes such as tumor location and cavities, into a logistic regression model to identify significant predictors of the MB subgroups. Similarly (31), extracted a set of 590 radiomic features from the tumor regions, including intensity-based histograms and local area integral invariant features, that were fed to a classifier to obtain the features that are prognosticative of the MB subgroups. The study in (30) employed a regional convolutional neural network to determine the molecular MB subgroups by utilizing prognosis information, masks confining only lesion areas, as well as genotyping information. These works reported significant differences among the MB subgroups yet have mainly focused on attributes from within the tumor confines without considering the tumor mass effect on the normal BAT area.

The analysis conducted in our work was limited to Gd-T1w images because the well-curated age-appropriate pediatric database (19) only included Gd-T1w MRI protocol for atlas construction. Unfortunately, corresponding age-appropriate atlases were not readily available for T2-w and FLAIR scans and hence these protocols could not be employed for our analysis. Interestingly, our deformation features captured from the BAT region showed significant differences among the different molecular subgroups of MB. Specifically, skewness of deformation (which indicates lack of data symmetry (i.e., heterogeneity)) emerged as a statistically significant feature between SHH and Group 3, and Group 3 and Group 4. The higher skewness values exhibited by the Group 3 at several distances from the tumor margin (**Figures 2B–D**) could be linked to the way such aggressive tumors proliferate and exert pressure on BAT, and hence may lead to a highly skewed distribution of the deformation magnitude values at these regions, compared to less aggressive, heterogeneous subgroups. Additionally, kurtosis was also statistically significant between Group 3 and Group 4, and SHH and Group 3. The higher values exhibited by Group 3 (**Figure 2A**) could be on account of the highly aggressive nature of this group, which leads to higher extremes in the deformation magnitude values associated with it. Our deformation descriptor could thus be part of an integrative analysis that explores the association of high-risk MB subgroups with dysregulated signaling pathways that are known to play important parts in the carcinogenesis or progression of MB (12). For example, it is known that PI3K/AKT pathway is activated during MB cell proliferation, which might be linked to the higher deformation magnitudes’ statistics values found on MB aggressive subtypes (e.g., Group 3 and Group 4), that exert pressure around the tumor. This pathway is also specifically related to poor prognosis in Group 3 and Group 4 (32, 33).

Apart from identifying MB subgroups, previous works in the context of MB survival analysis have investigated clinical and biological predictors or gene expression profiling analysis (34, 35). More recently, one study developed a radiomic signature for survival analysis in MB using a combination of shape features extracted from the tumor contour, as well as intensity and texture features extracted from both the original images and the Laplacian-transformed images (12). This study further extended their analysis to obtain dysregulated pathways across the low-, and high-risk categories obtained from the radiomic signature. Additionally, the study demonstrated that a combined radiomics-clinical-molecular signature improved survival risk-stratification as compared to employing radiomic features alone. These results are in line with our findings in this work, which suggest that the radiomic attributes obtained from imaging (represented in our deformation descriptor) may serve as a complementary tool to the current clinical approaches used in risk assessment, towards more reliable risk stratification in pediatric medulloblastoma.

In the context of our presented survival analysis, kurtosis and median of the deformation descriptor were identified as top features by the LASSO regression model, on the training cohort. Interestingly, kurtosis of deformation at 10 mm was statistically significantly different between high- and low-risk survival categories (**Figure 6**), with higher values for the high-risk survival group (as visually detected on **Figure 7**). This could be on account of the higher heterogeneity of deformations within the BAT regions in patients with poor survival, due to the structural distortions beyond tumor confines.

Additionally, when combining our deformation descriptor with the current risk assessment approaches that are used in a clinical setting (molecular subgroup information, Chang’s stratification), substantially improved performance metrics (i.e., C-index) were obtained for survival risk-assessment, compared to any of these three approaches in a univariate setting. This demonstrates the added value of combining the proposed radiomic descriptor with the current stratification approaches, towards more reliable risk assessment in pediatric MB.

Our study did have some limitations. Our results were limited to identifying significant associations of deformation features with molecular subgroups and overall survival using a relatively small cohort of retrospective studies (from 2 institutions) and will need to be validated on a larger multi-institutional cohort. Further, when employing the RRS survival model, either alone, or combined with the current clinical stratification approaches (molecular subgroup information and Chang’s classification system), significant differences were not observed between the two risk groups, on the test set, on account of the limited sample size (n = 28 for models involving RRS or RRS + Chang’s and n = 21 for the models that involved molecular information). In fact, it is well documented that larger sample sizes are required for a multivariate regression model to fit well to the data (~10-15 cases per covariate is recommended) (36), which was unfortunately not available with our limited cohort size in this study. Interestingly, our analyses also revealed that known prognostic markers (EOR, presence of metastatic disease) as well as clinical risk-stratification approaches (i.e., Chang and molecular stratification), in a uni-variate setting, were independently not prognostic of survival. While some works in the literature have reported similar findings and varied outcomes using these stratification approaches (10, 37, 38), we plan to further investigate the efficacy of these approaches on larger cohorts in the future. Another limitation was that the mutation information related to the sub-stratification of the 4 molecular subgroups was not available for our cohorts, e.g., the sub-stratification of SHH subgroup for tp53 mutation and MYCN amplification. Hence, additional analyses relating patient prognosis to mutation-specific sub-stratification could not be performed. Lastly, the lack of uniformity in the treatment strategies applied to the different MB subgroups might be a confounder and was not specifically accounted for in our survival analysis.

Our future work will aim to apply our proposed deformation descriptor to a group of uniformly treated patients with pediatric medulloblastoma. In addition, a larger cohort that evaluates progression free survival (along with overall survival) will be employed, to further demonstrate the importance of RRS, complementary to, and in conjunction with existing clinical prognostic markers (EOR. presence of metastasis), as well as Chang and molecular stratification. Also, we aim to integrate deformation descriptors with radiomic texture and shape features from the tumor regions using multi-parametric MRI sequences (T2w, FLAIR, perfusion) for molecular and survival risk-stratification. Further, to ensure clinical utility of our approaches, we aim to build an automated segmentation pipeline for pediatric brain tumors, utilizing our currently curated dataset that includes carefully annotated tumor sub-compartments by expert neuro-radiologists. We also will seek to investigate other clinical attributes as manifested on imaging, such as CSF seeding, and incorporating them as complementary features to our deformation descriptor, for MB survival prognostication and subgroup stratification. Lastly, we will seek to include pathway-specific information in our curated studies, to establish associations between signaling pathways and our deformation descriptor in MB tumors.

To conclude, in this preliminary feasibility study, we identified associations between our local tissue deformation features extracted from routine Gd-T_1w_ protocol with overall survival in MB patients. Following rigorous prospective multi-site evaluation, the deformation features associated with molecular subtypes could provide complementary information beyond molecular and histological phenotypes towards reliable survival risk stratification and help guide therapeutic treatment management in MB patients.

## Data availability statement

The raw data supporting the conclusions of this article will be made available by the authors, without undue reservation.

## Author contributions

SI contributed to analysis and interpretation of data, contributed to designing the experiments and the software, helped drafting the original article. MI contributed to the conceptualization, analysis, experimental design and software, and interpretation of data, wrote the original draft and continued revising the article based on co-authors’ feedback. BT provided clinical datasets, led the conceptualization, and helped define the clinical problem and provided clinical interpretation of findings. RS provided clinical datasets, supported the conceptualization, and helped define the clinical problem and provided clinical interpretation of findings. PB and ASM helped curating the clinical datasets and helped with clinical interpretation of findings. RC and JC helped curating the clinical datasets and performed the annotations on radiological images. KB performed the annotations on radiological images. VS supervised all the annotations and corrected them. M-LH supported the conceptualization of the clinical problem. PV and RV contributed to the analysis. DH and AJ helped curating the clinical datasets and helped with clinical interpretation of findings. AM revised the manuscript critically for important intellectual content. PT contributed to the interpretation of the results, contributed to designing the experiments and conceptualization, and supervised the writing and edited the article. PF helped with the design of all the conducted statistical tests. All authors have reviewed the manuscript. All authors read and approved the final manuscript.
