# Erratum: Novel MRI deformation-heterogeneity radiomic features are associated with molecular subgroups and overall survival in pediatric medulloblastoma: Preliminary findings from a multi-institutional study

**DOI:** 10.3389/fonc.2023.1163188

**Published:** 2023-02-20

**Authors:** Frontiers Production Office

**Affiliations:** Frontiers Media SA, Lausanne, Switzerland

**Keywords:** medulloblastoma, deformation, molecular subgroups, survival, LASSO

Due to a production error, there were errors in the affiliations list. Instead of “Department of Population and Quantitative Health Sciences, Case Western Reserve University, Cleveland, OH, United States”, affiliation 1 should have been “Department of Biomedical Engineering, Case Western Reserve University, Cleveland, OH, United States”. Furthermore, instead of “Department of Radiology, University of Wisconsin-Madison, Madison, WI, United States”, affiliation 2 should have been “Department of Radiology and Biomedical Engineering, University of Wisconsin-Madison, Madison, WI, United States”.

In addition, the author Pingfu Fu’s affiliation was listed incorrectly. Instead of being associated with affiliation 1, a new affiliation should have been added. The new affiliation listed for Pingfu Fu is “8 Department of Population and Quantitative Health Sciences, Case Western Reserve University, Cleveland, OH, United States”.

The publisher apologizes for these mistakes. The original version of this article has been updated.

